# Supplementary material for: Determinants of functional synaptic connectivity among amygdala-projecting prefrontal cortical neurons in male mice
Source: Nat Commun. 2023 Mar 25;14:1667. doi: 10.1038/s41467-023-37318-x (PMC10039875; doi:10.1038/s41467-023-37318-x)
Supplement: Supplementary file 1 — Supplementary Information [file 41467_2023_37318_MOESM1_ESM.pdf]

Supplementary data

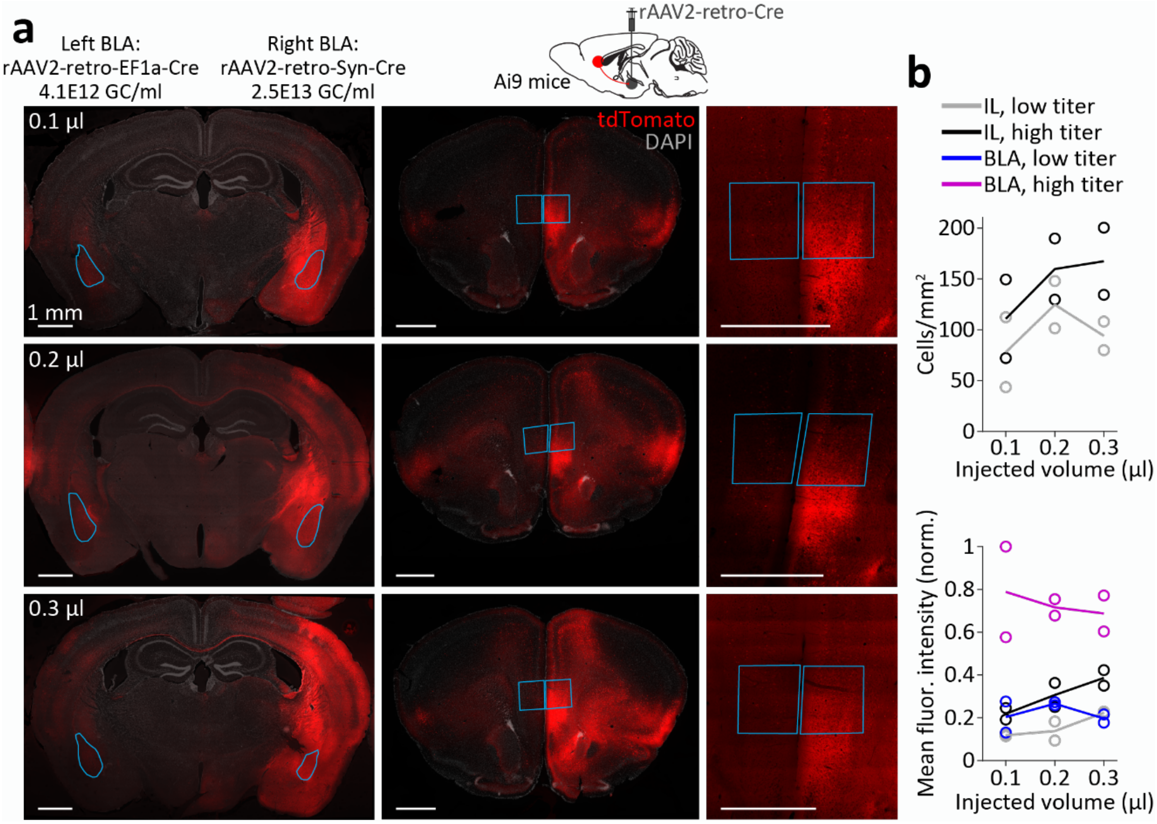

Figure S1. Retrograde labeling of cells projecting to the BLA. a–b, Injection of Cre-expressing rAAV2-retro vectors into the BLA of Ai9 mice, which contain a Cre-dependent tdTomato expression cassette. Notice that rAAV2-retro-Cre leads to retrograde expression of Cre (and thereby tdTomato) in cells at presynaptic sites as well as at the injection site (BLA). This leads to fluorescent cell bodies in the BLA and to dense fluorescent axonal fibers at the mPFC (as well as retrograde cell bodies) which originate at the BLA. See Methods for details on the specificity of retrograde labeling from the BLA. **a**, Coronal sections (40 µm thickness) containing the BLA (left) and the mPFC (middle and right) of three different mice, each injected with a different volume of the viral vector (top, 100 nl at injection rate of 10 nl/min; middle, 200 nl at 20 nl/min; bottom, 300 at 30 nl/min). The left BLA of each mouse was injected with rAAV2-retro-EF1α-Cre with titer of 4.1×10^{12} genome copies per milliliter (gc/ml), and the right BLA was injected with the same volume of rAAV2-retro-hSyn-Cre with titer of 2.5×10^{13} gc/ml. Images on the right are high-resolution confocal scans of the mPFC of the same mice as the images in the middle. All scale bars are 1 mm. Sagittal atlas illustration on top was adapted from ref. 107. **b**, Quantification of cell density (top) and mean fluorescence intensity (bottom) in the mPFC and in the BLA, as a function of injected AAV volume and its titer. *n* = 2 mice for each volume. Circles represent individual mice and lines represent means. Measured regions are marked in cyan in **a** for three of the mice. Cell density in the BLA was not measured due to the high density of axonal fibers originating from retrogradely labeled presynaptic sites. Source data are provided as a Source Data file.

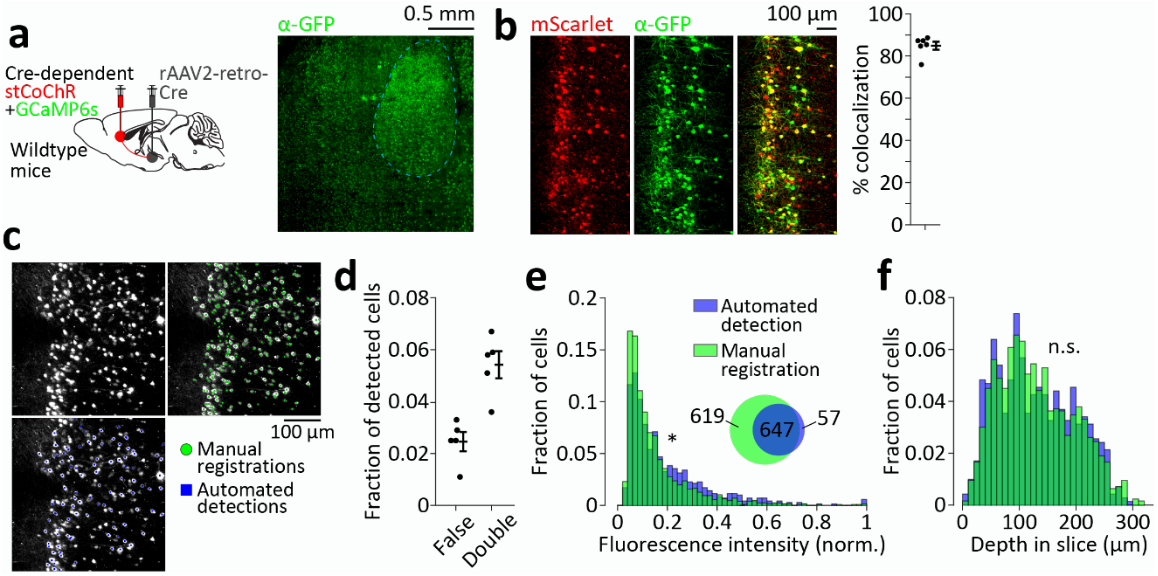

Figure S2. Retrograde labeling of mPFC-BLA cells and automated detection of cell bodies. a–f, Injection of rAAV2-retro-Cre into the BLA and Cre-dependent stCoChR and GCaMP6s into the mPFC of wildtype (C57BL/6J) mice to label mPFC-BLA cells. **a**, mPFC-BLA cell labeling strategy (left, sagittal atlas illustration was adapted from ref. 107) and a confocal z-projection image showing axonal labeling in the BLA, visualized using anti-GFP immunostaining to amplify the GCaMP6s fluorescence (right, BLA outlined in dashed cyan). **b**, Confocal images showing expression of mScarlet (co-expressed with stCoChR) and GCaMP6s (amplified using anti-GFP immunostaining) in mPFC-BLA cells (left), and quantification of the co-expression of mScarlet and GCaMP6s in mPFC-BLA cells based on $n = 6$ images from two mice (right). Co-expression is calculated as the fraction of cells expressing both mScarlet and GCaMP6s out of the total cells expressing either, within an area of 0.5×1 mm^2 containing the densest expression in the infralimbic cortex. Error bars indicate mean ± SEM. **c**, An example two-photon z-projection image used for connectivity mapping (top left), with overlaid labeling of cell bodies registered manually (green, right) and cell bodies detected with the automated algorithm (blue, bottom). **d–f**, Performance of the algorithm for automated detection of cell bodies in a scanned tissue volume. **d**, Fraction of false detections (where detection does not point to a cell) and double detections (where detection points between two adjacent cells) out of a total of 1046 automated detections in $n = 5$ scanned volumes from two mice. Error bars indicate mean ± SEM. **e**, Distribution of fluorescence intensity across automatically detected cells ($n = 704$) and manually registered cells ($n = 1266$) in four scanned volumes from four mice. * One-tailed, two-sample Kolmogorov-Smirnov test for manually vs. automatically detected cells: $D_{1266,704} = 0.14$, $p = 2.2 \times 10^{-8}$. All intensities are normalized to the maximal fluorescence among all cells within the same volume. Bin size is 0.02. Inset shows a Venn diagram of the manually registered and automatically detected cell bodies. **f**, Distribution of cell positions along the depth of the slice for the same cells as in **e**. Two-tailed, two-sample Kolmogorov-Smirnov test: $D_{1266,704} = 0.04$, $p = 0.49$. Bin size is 10 µm. Source data are provided as a Source Data file.

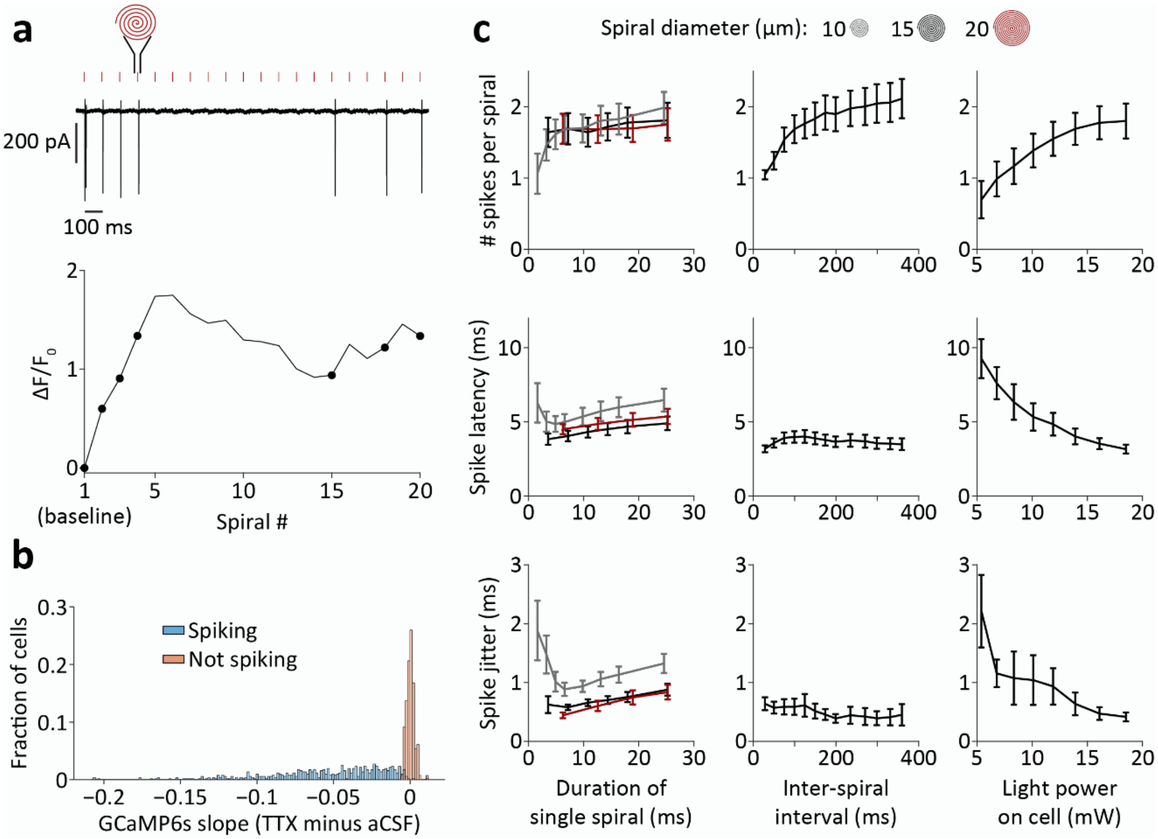

Figure S3. Spiral stimulation parameters and detection of spiking using GCaMP6s. a, Cell-attached recording (top) and GCaMP6s $\Delta F/F_0$ (bottom) from an mPFC-BLA cell during a train of 20 spirals (3.56 ms each) delivered at 10 Hz. Red ticks denote spiral-scan periods, and spike-triggering spirals are highlighted by circles over the $\Delta F/F_0$ trace. **b**, Distribution of the difference between the GCaMP6s fluorescence slope in presence of TTX and its slope in absence of TTX, based on the cells in Figure 1h. The distribution is presented separately for cells that were determined to have spiked (blue) and those that did not spike (red) based on the lower bound of the confidence interval of the GCaMP6s fluorescence slope. Bins are 0.0015. **c**, Cell-attached recordings from mPFC-BLA cells expressing stCoChR and GCaMP6s during scanning with trains of 10 spirals. Diameter and duration of each spiral (left; *n* = 11 cells; light power on cell = 13.9 mW, inter-spiral interval = 100 ms), time interval between consecutive spirals (middle; *n* = 11 cells; light power on cell = 13.9 mW, spiral duration = 3.6 ms), and light power on cell (right; *n* = 10 cells; spiral duration = 3.6 ms, inter-spiral interval = 100 ms) were varied, while the number of evoked spikes per spiral (top), latency to first spike (middle), and jitter of first spike (bottom) were measured. Error bars indicate mean ± SEM. See Figure 1i for additional measurements. Source data are provided as a Source Data file.

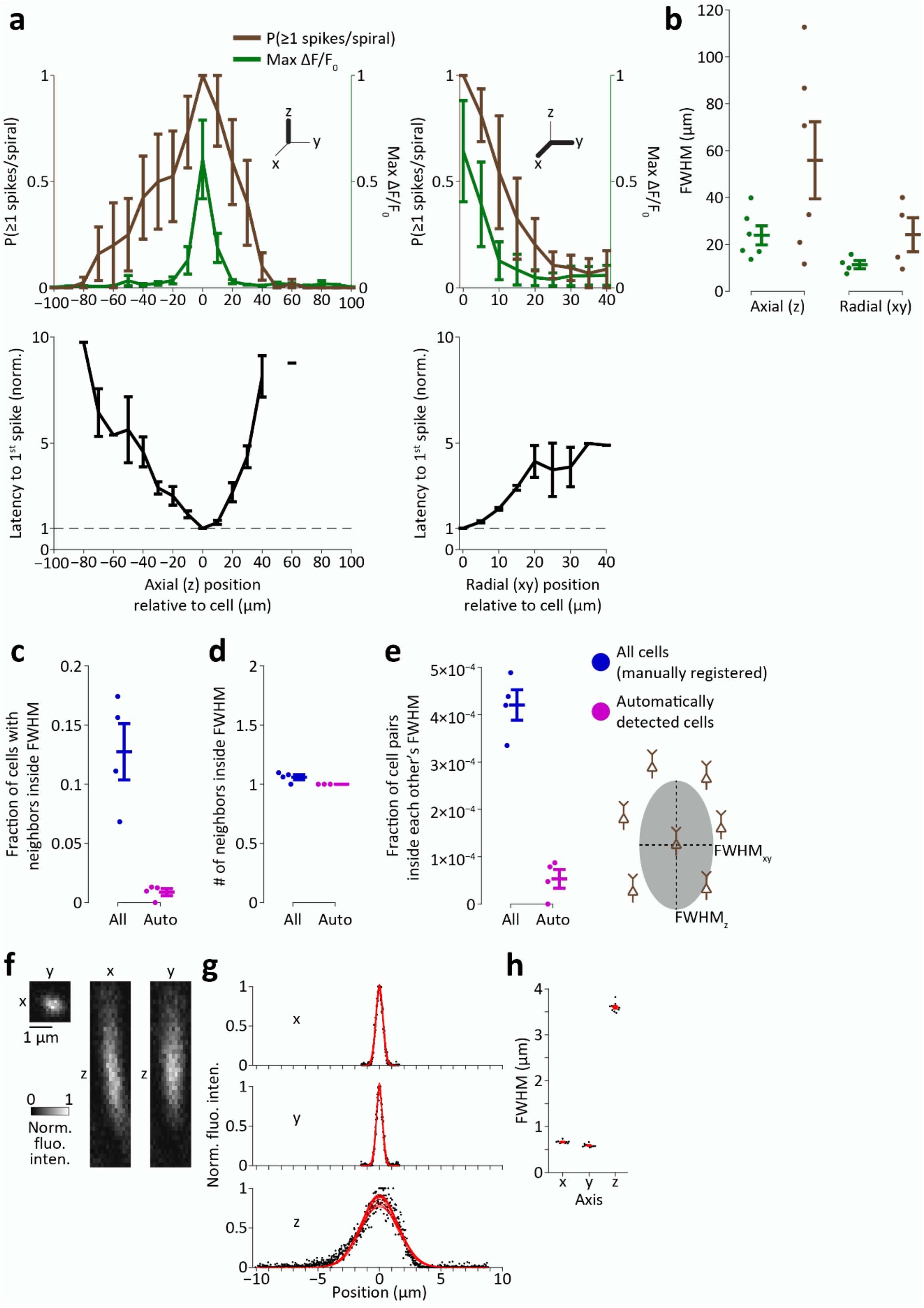
a
P(≥1 spikes/spiral)
Max ΔF/F0
P(≥1 spikes/spiral)
Max ΔF/F0
Latency to 1st spike (norm.)
Axial (z) position relative to cell (µm)
Radial (xy) position relative to cell (µm)
b
FWHM (µm)
Axial (z)
Radial (xy)
c
Fraction of cells with neighbors inside FWHM
All
Auto
d
# of neighbors inside FWHM
e
Fraction of cell pairs inside each other's FWHM
All cells (manually registered)
Automatically detected cells
FWHMxy
FWHMz
f
1 µm
Norm. fluo. inten.
g
Norm. fluo. inten.
Position (µm)
h
FWHM (µm)
Axis

Figure S4. Spatial specificity of stimulation and imaging relative to the labeled-cell density in the slice preparation. a, Spatial specificity of stimulation and imaging measured using cell-attached recordings from mPFC neurons while spiral patterns were scanned at varying positions relative to the cell (light power 10 mW in focus). Left, Specificity across focal planes (axial, z). Right, Specificity within the focal plane (radial, xy). Top, Probability for evoking at least one spike per spiral (brown) and maximal $\Delta F/F_0$ in a spiral train (green). Bottom, Latency to first spike, normalized to the latency on the cell. n = 6 cells for axial specificity and n = 4 cells for radial specificity. Here and throughout this figure, error bars indicate mean ± SEM. **b**, Full width at half maximum (FWHM) for axial specificity (left, z) and for radial specificity (right, xy), based on individual-cell data in **a**. FWHM of spiking vs. $\Delta F/F_0$: Wilcoxon signed rank test, $p = 0.44$ in z and $p = 0.12$ in xy. **c**, Fraction of cells in a scanned tissue volume which have at least one neighboring cell that is found within their spiking FWHM ellipsoid. Data is presented for all cells in a volume as registered manually (blue) and for cells detected automatically using the soma-detection algorithm (purple). n = 4 scanned volumes from four mice. **d**, Mean number of within-FWHM-ellipsoid neighbors, among the cells having within-ellipsoid neighbors, in the same scanned volume as in **c**. **e**, Fraction of cell pairs, among all possible pairs in a volume, which reside inside each other's FWHM ellipsoid, in the same scanned volume as in **c**. Bottom right, Diagram describing the cell-density calculations with respect to the FWHM ellipsoid. **f–h**, Point spread function of the two-photon microscope system used in this study. **f**, Cross-section images of an example fluorescent microsphere across three planes. **g**, Fluorescence intensity as a function of the two-photon beam position relative to the microsphere center, across the three microscope axes for n = 9 microspheres. Gaussian fits for each individual microsphere are presented in red. **h**, FWHM of the fluorescence intensity at each axis, based on the Gaussian fits in **g**. Source data are provided as a Source Data file.

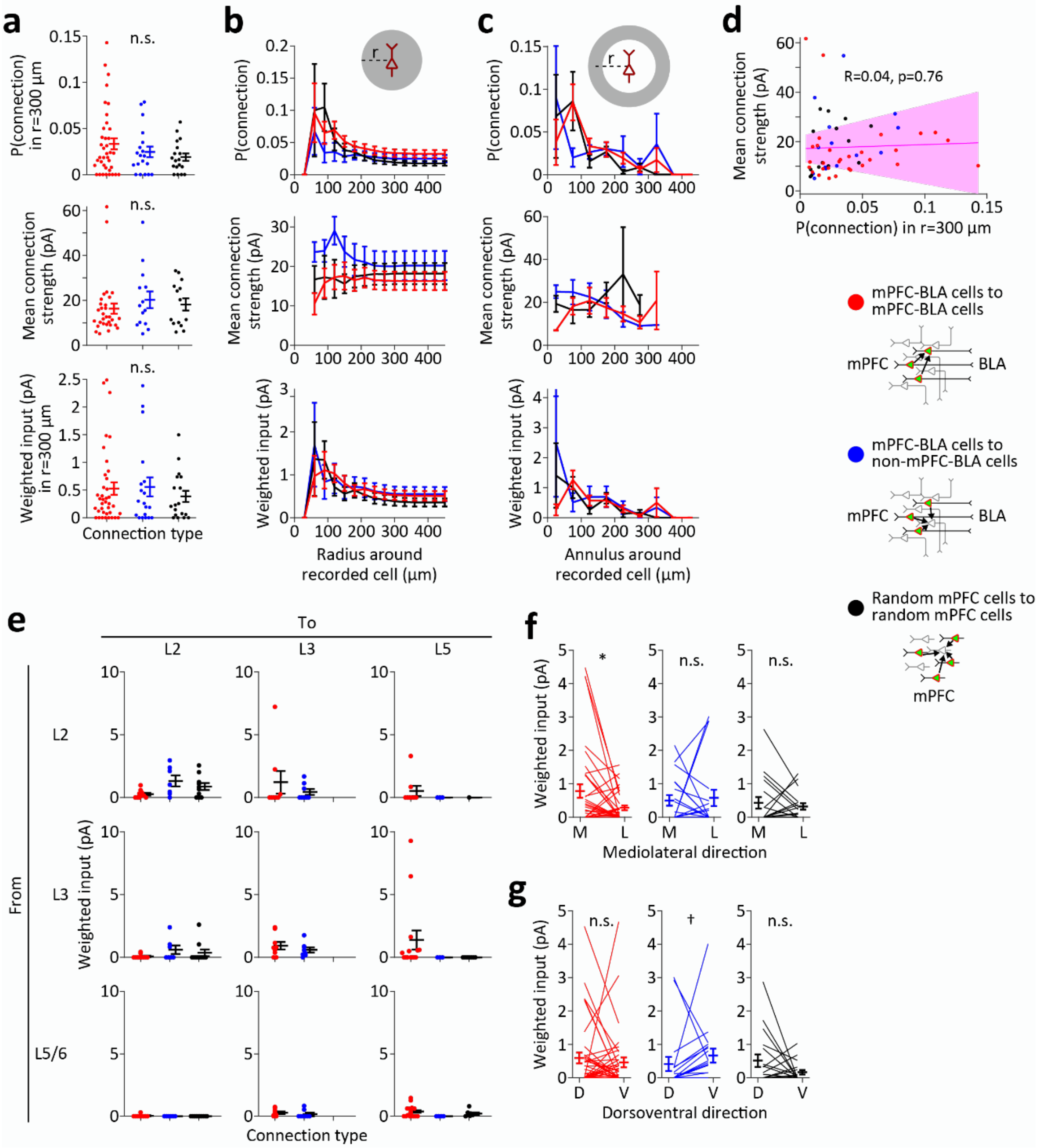

Figure S5. Anatomical distribution of synaptic connections. **a**, Overall probability of connection within a distance of 300 µm from the recorded cell (top), mean connection strength (middle, with no restriction to the distance from the recorded cell), and weighted input onto the recorded cell within a 300 µm distance (bottom), for each connection type. Points are individual recorded cells corresponding to single maps. Here and throughout this figure, error bars indicate mean ± SEM. Zero probability of connection (and, accordingly, zero weighted input) indicates that the recorded cell did not receive input from the stimulated cells. Kruskal-Wallis test with multiple post hoc comparisons using Tukey's HSD, n.s. $p \geq 0.52$. For **a–c**: $n = 38$ cells in mPFC-BLA to mPFC-BLA connections, $n = 19$ cells in mPFC-BLA to non-mPFC-BLA connections, and $n = 18$ cells in random mPFC to random mPFC connections. **b**, Probability of connection (top), connection strength (middle), and weighted input (bottom) as function of the radius of the sphere around the recorded cell where connections are measured. **c**, Probability of connection (top), connection

strength (middle), and weighted input (bottom) as function of the center of the annulus (50 μm thickness) around the recorded cell where connections are measured. **d**, Lack of correlation between the probability of connection and connection strength across all connection types (n = 38 cells in mPFC-BLA to mPFC-BLA connections, n = 19 cells in mPFC-BLA to non-mPFC-BLA connections, and n = 18 cells in random mPFC to random mPFC connections). The line and the shaded region represent the regression line and its 95% confidence bounds, respectively. **e**, Summary of cross-layer connections measured by weighted input. Notice that stimulated (presynaptic) cells in layers 5 and 6 were pooled. n cells are detailed in Figure 3d. **f**, Weighted synaptic input as function of mediolateral position of the stimulated cells relative to the postsynaptic recorded cell. n = 38 cells in mPFC-BLA to mPFC-BLA connections, n = 19 cells in mPFC-BLA to non-mPFC-BLA connections, and n = 18 cells in random mPFC to random mPFC connections. Wilcoxon signed rank test, * $p = 0.029$; n.s. $p \geq 0.67$. **g**, Weighted synaptic input as function of dorsoventral position relative to the recorded cell. n cells as in **f**. Wilcoxon signed rank test, † $p = 0.091$; n.s. $p \geq 0.22$. Source data are provided as a Source Data file.

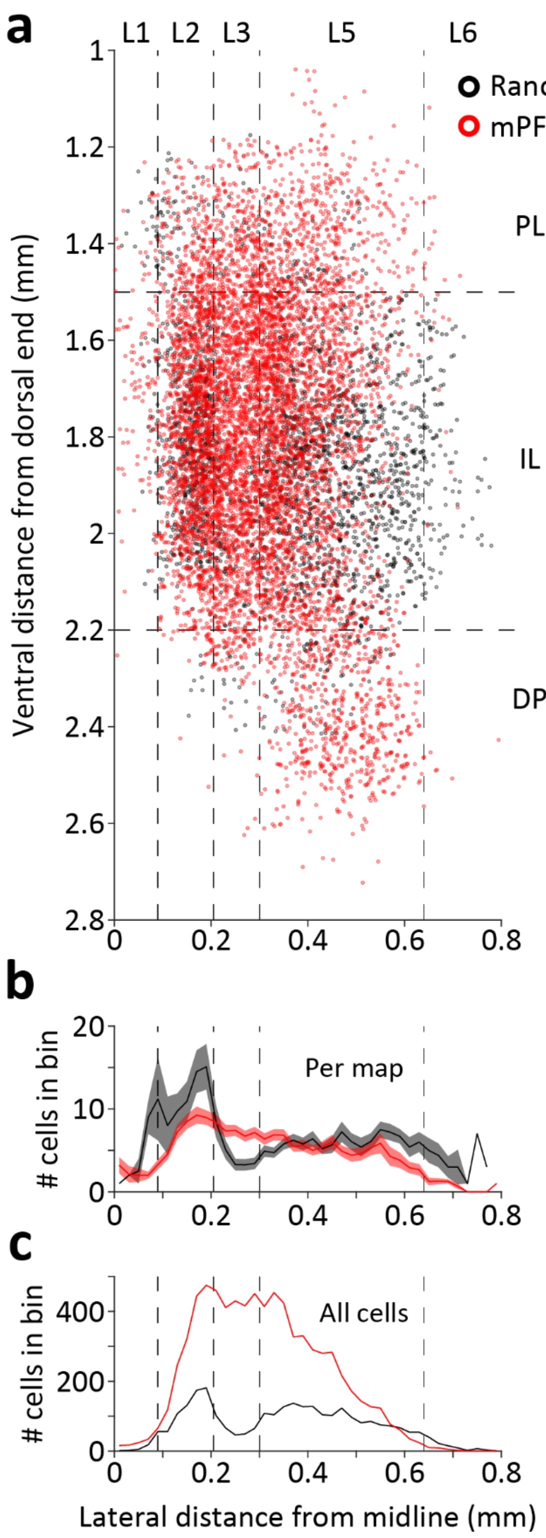

Figure S6. Distribution of stimulated-cell positions in anatomical space. a, Positions of all cells that were stimulated during recording from corresponding cells located throughout the mPFC (PL, IL, and DP), in dorsoventral (DV) and mediolateral (ML) coordinates. mPFC-BLA cells are in red (n = 7737 cells from 67 experiments corresponding to 67 recorded cells), and randomly labeled mPFC cells are in black (n = 2802 cells from 22 experiments corresponding to 22 recorded cells). Vertical dashed lines indicate division into layers, based on cell density[107] shown in **b** (layer borders: 90, 205, 300, and 640 μm from midline[75]). L1 contains excitatory projection cells, consistent with known presence of L2 cells in L1 of the mPFC[107]. Horizontal dashed lines indicate division into subregions (PL/IL border: 1.5 mm; IL/DP border: 2.2 mm from dorsal midline edge). Notice that the anteroposterior (AP) position was collapsed (range of AP positions in all experiments: 1.2 to 2 mm from bregma), such that the IL/PL/DP borders are not consistent across experiments and are drawn based on average AP position. Borders of L3 were determined based on local reduction in cell density in the random-cell class[107], and layer borders were consistent with marker expression data[75]. **b**, Number of cells in each experiment in 20 μm bins along the ML axis. Shaded areas indicate SEM. **c**, Total number of cells in all experiments in 20 μm bins. Source data are provided as a Source Data file.

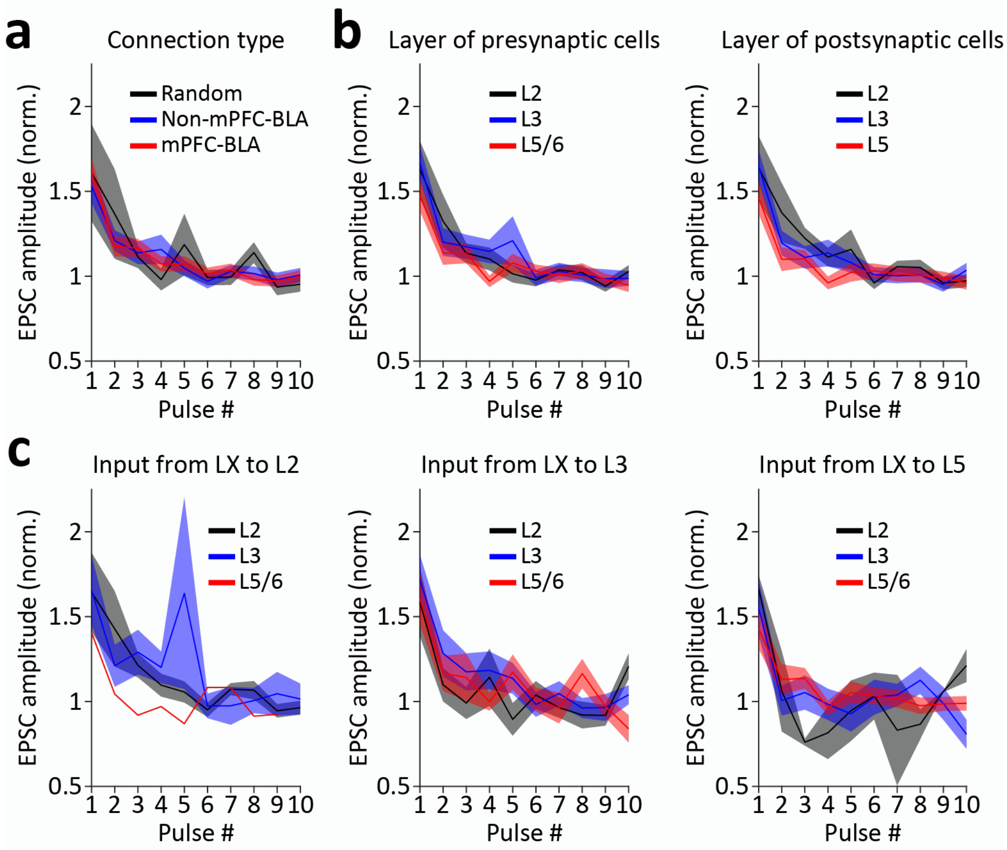

Figure S7. Short-term plasticity of synaptic connections. a, Amplitude of evoked EPSC as function of the stimulation pulse, normalized to the mean of the last five pulses, for the three connection types as in Figure 3a. Only the first 10 pulses were considered in experiments with a 15-pulse train, and in experiments with multiple repetitions of the stimulation protocol, all repetitions were averaged. Here and throughout this figure, lines are means and shaded regions represent SEM. **b**, Same as **a**, but pooling all connection types and separating according to the layer of the presynaptic cells (left) or layer of the postsynaptic cells (right). **c**, Same as **b**, but pooled according to both the presynaptic and the postsynaptic cell layer. Source data are provided as a Source Data file.

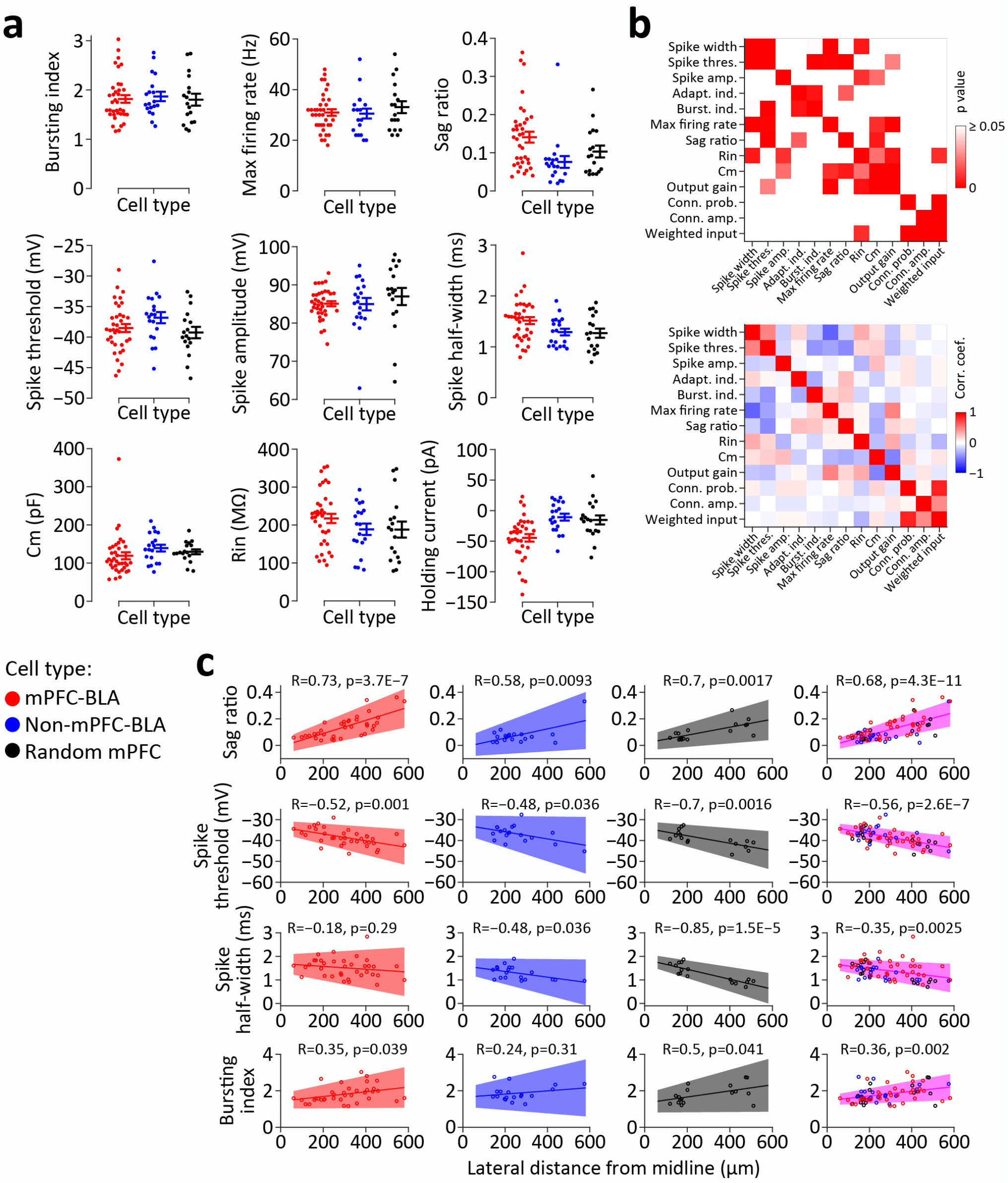

Figure S8. Electrophysiological properties of pyramidal neuron populations in the ventral mPFC. a, Electrophysiological properties of the different cell types. Color code as in Figure 4. Error bars indicate mean ± SEM. See Figure 4b–e for additional properties. Notice that the apparent difference in sag ratio between the cell types stems from this parameter's correlation with mediolateral position (**c**) and the larger fraction of mPFC-BLA cells in L5 compared with the other cell types (Table S1). **b**, Pairwise correlations between electrophysiological properties and connectivity properties. Top, P values of pairwise Pearson correlations. Bottom, Correlation coefficients for pairwise Pearson correlations. Connection probability, connection amplitude, and weighted input are calculated as in Figure S5a. **c**, Correlation between cells' mediolateral position and their electrophysiological properties, for each cell type separately (three left columns) and for all cells pooled (right column). In each plot, each point represents a cell, the line is a linear fit, and the shaded region represents the 95% confidence intervals of the linear fit. Magenta (right column) represents fits for all cells pooled (individual cells are color-coded by type). Source data are provided as a Source Data file.

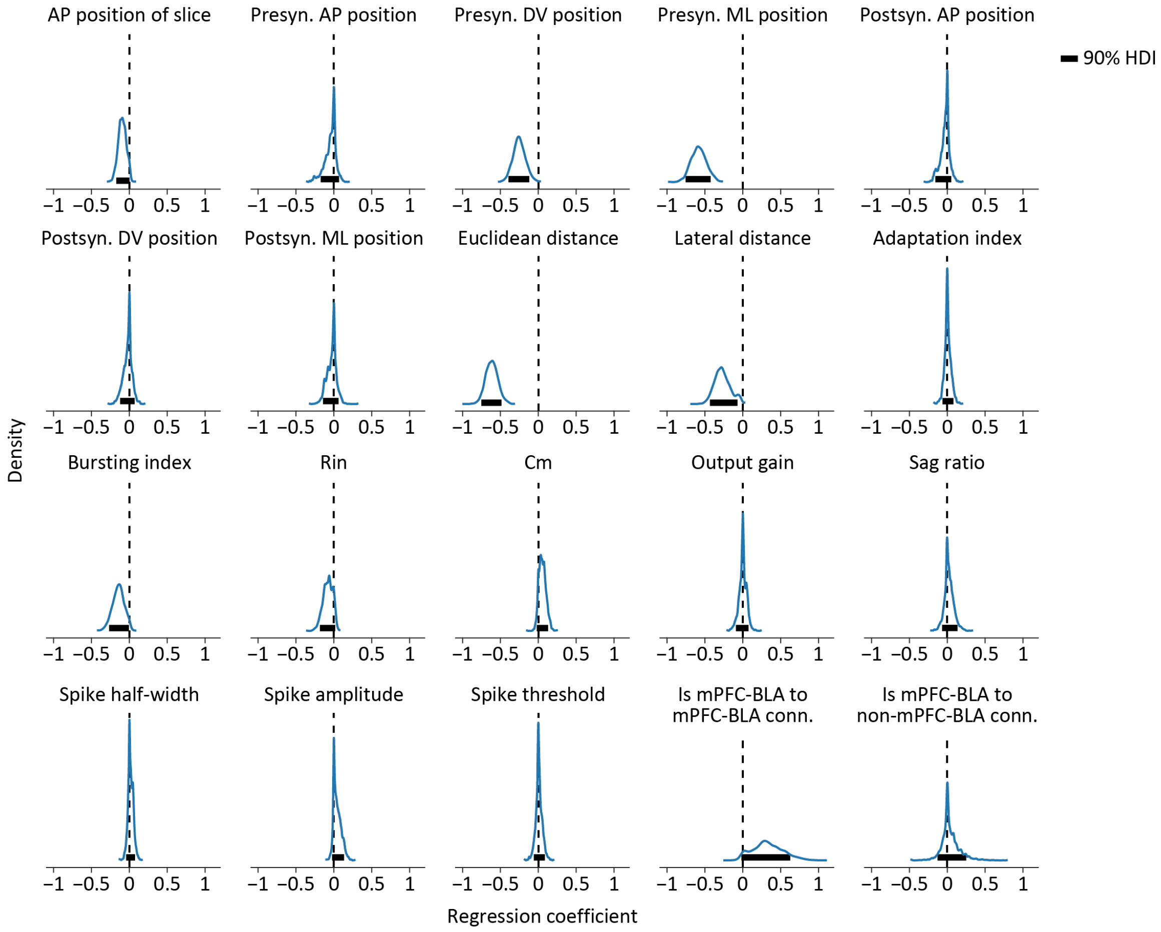

Figure S9. Posterior distributions of regression coefficients calculated using Horseshoe priors. Thick horizontal lines represent the 90% highest density interval (HDI). Density scaling is identical for all features. See Figure 4h for summary of the distributions.

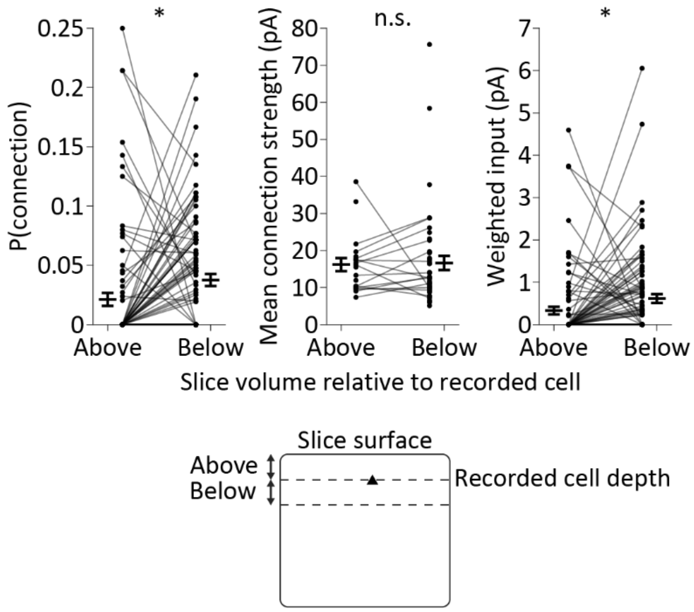

Figure S10. Connectivity properties arising from the slice volume located above the recorded cell and from an equally sized volume below the recorded cell. n = 92 cells of all types in the entire mPFC. Wilcoxon signed rank test, * p = 0.006; n.s. p = 0.30. Error bars indicate mean ± SEM. Source data are provided as a Source Data file.

Table S1. Total numbers of cells and animals in each experiment type.

			Map type		
			mPFC-BLA to mPFC-BLA	mPFC-BLA to non-mPFC-BLA	Random to random
Number of postsynaptic cells (maps)	Ventral mPFC (IL+DP)	L2	10	7	10
		L3	9	8	0
		L5	19	4	8
		L6	0	0	0
	Dorsal mPFC (PL+Cg)	All layers	9	2	6
Number of stimulated cells (responsive) *, **	Ventral mPFC (IL+DP)	L2	883	586	1169
		L3	1161	846	0
		L5	1439	500	913
	Dorsal mPFC (PL+Cg)	All layers	659	150	474
Number of animals ***	Ventral mPFC (IL+DP)	L2	7	6	4
		L3	8	5	0
		L5	14	3	6
	Dorsal mPFC (PL+Cg)	All layers	8	2	4

* In this Table, all stimulated cells within a given map are considered to be in the same layer as the postsynaptic cell of the same map.

** Only stimulated cells determined to have spiked in response to stimulation by their GCaMP6s signal are counted.

*** The same animal can be counted multiple times when it was used for recording from cells in different regions or from different cell types (mPFC-BLA cells and non-mPFC-BLA cells). n = 38 animals were used in total for recording in this study.

Table S2. Numbers of cells per each individual experiment.

Map type	Recorded (postsynaptic) cell		Stimulated cells			
	Region	Layer	# total	# spiking	# included *	% spiking
mPFC-BLA to mPFC-BLA	IL	5	45	35	34	77.8
	IL	2	204	143	138	70.1
	IL	3	160	141	129	88.1
	IL	3	116	89	81	76.7
	IL	3	246	242	228	98.4
	IL	3	179	159	154	88.8
	DP	5	32	13	13	40.6
	DP	5	236	234	233	99.2
	PL	5	245	215	112	87.8
	PL	5	169	72	70	42.6
	IL	5	118	104	101	88.1
	PL	5	79	67	65	84.8
	IL	5	94	83	81	88.3
	PL	3	63	21	20	33.3
	IL	2	152	130	128	85.5
	PL	2	89	49	48	55.1
	PL	5	41	21	21	51.2
	IL	3	186	166	162	89.2
	IL	5	175	153	152	87.4
	PL	3	117	97	95	82.9
	PL	2	77	61	61	79.2
	DP	5	179	136	131	76.0
	IL	3	118	111	106	94.1
	IL	5	88	56	56	63.6
	PL	3	93	56	54	60.2
	IL	2	94	74	70	78.7
	IL	5	56	43	43	76.8
	IL	2	94	82	81	87.2
	IL	2	66	54	54	81.8
	IL	5	95	45	44	47.4
	IL	5	59	26	20	44.1
	IL	5	64	42	40	65.6
	IL	3	76	50	44	65.8
	DP	5	90	63	47	70.0
	DP	5	119	67	67	56.3

	IL	3	126	121	108	96.0
	IL	5	89	63	58	70.8
	IL	5	80	51	50	63.8
	IL	2	40	31	31	77.5
	IL	2	78	76	71	97.4
	IL	2	103	87	78	84.5
	IL	2	111	96	84	86.5
	IL	2	145	110	109	75.9
	IL	3	113	82	79	72.6
	IL	5	118	101	101	85.6
	IL	5	134	93	92	69.4
	IL	5	47	31	31	66.0
mPFC-BLA to non-mPFC-BLA	Cg	5	118	88	88	74.6
	IL	3	246	225	224	91.5
	IL	2	148	70	68	47.3
	IL	5	211	194	194	91.9
	IL	5	113	106	106	93.8
	IL	3	100	88	88	88.0
	IL	3	93	79	78	84.9
	IL	2	110	86	86	78.2
	IL	2	66	35	35	53.0
	DP	5	181	136	136	75.1
	IL	2	101	90	90	89.1
	IL	2	124	106	106	85.5
	IL	2	135	110	110	81.5
	IL	5	70	64	64	91.4
	IL	3	114	77	77	67.5
	IL	3	151	131	130	86.8
	IL	3	109	102	101	93.6
	IL	3	104	99	99	95.2
	IL	2	100	89	88	89.0
	PL	3	92	62	62	67.4
	IL	3	71	45	45	63.4
Random to random	IL	5	155	143	141	92.3
	Cg	5	91	78	78	85.7
	IL	5	106	105	104	99.1
	IL	5	106	104	102	98.1
	IL	5	156	137	135	87.8
	PL	5	109	63	63	57.8

	PL	5	142	129	129	90.8
	PL	5	50	43	42	86.0
	PL	5	126	115	114	91.3
	IL	5	151	132	131	87.4
	IL	5	163	143	142	87.7
	IL	5	129	109	109	84.5
	IL	2	104	90	88	86.5
	IL	5	117	40	40	34.2
	Cg	2	69	46	46	66.7
	IL	2	201	193	161	96.0
	IL	2	148	144	142	97.3
	IL	2	158	152	138	96.2
	IL	2	93	72	70	77.4
	IL	2	73	57	57	78.1
	IL	2	176	170	166	96.6
	IL	2	85	68	63	80.0
	IL	2	157	147	121	93.6
	IL	2	97	76	75	78.4

* Cells included in the analysis, after exclusion of all stimulated cells according to criteria detailed in Methods.

Table S3. Statistics for electrophysiological properties of all cell types.

Property	Cell type									Kruskal-Wallis test		Post hoc comparisons (*p* value)		
	mPFC-BLA			Non-mPFC-BLA			Random mPFC			χ^2	*p* value	mPFC-BLA vs. non-mPFC-BLA	mPFC-BLA vs. random mPFC	Non-mPFC-BLA vs. random mPFC
	n	Mean	SEM	*n*	Mean	SEM	*n*	Mean	SEM					
Rm (MΩ)	36	217.4	12.3	19	188.8	15.0	17	188.1	21.1	2.7	0.26	0.49	0.29	0.93
Cm (pF)	36	119.2	9.2	19	139.2	8.8	17	129.6	6.5	6.0	0.051	0.063	0.23	0.89
Max firing rate (Hz)	36	30.9	1.3	19	30.5	1.9	17	33.1	2.4	0.56	0.75	0.97	0.82	0.75
Output gain (Hz/pA)	36	0.13	0.0077	19	0.10	0.005	17	0.12	0.010	7.8	0.020	0.015	0.63	0.26
Adaptation index	36	3.3	0.21	19	1.95	0.16	17	2.3	0.20	24.1	5.8×10^{-6}	6.0×10^{-6}	0.016	0.24
Bursting index	36	1.82	0.078	19	1.87	0.095	17	1.80	0.12	0.69	0.71	0.74	0.99	0.74
Sag ratio	36	0.14	0.014	19	0.076	0.015	17	0.10	0.016	11.7	0.0029	0.0021	0.25	0.31
Spike threshold (mV)	36	–38.5	0.66	19	–36.8	0.91	17	–39.3	0.93	3.9	0.14	0.24	0.86	0.16
Spike half-width (ms)	36	1.52	0.069	19	1.29	0.065	17	1.27	0.09	6.3	0.044	0.10	0.10	1.00
Spike amplitude (mV)	36	85.1	0.66	19	85.0	1.6	17	87.0	2.2	3.3	0.19	0.94	0.17	0.39
Holding current (pA)	34	–44.6	6.2	19	–10.7	5.7	18	–15.2	7.4	15.2	5.0×10^{-4}	0.0013	0.014	0.83

The Kruskal-Wallis test with multiple post hoc comparisons using Tukey's Honestly Significant Difference (HSD) procedure was used for comparing electrophysiological properties across cell types.

Table S4. Parameters used for detection and modeling of EPSCs.

Parameter	Value	Description
Detrending parameters		
f_sample	5000 Hz	Frequency to which the signal was downsampled (using the resample_poly function)
order_filter_len	50 ms	Sliding window of the 10th percentile filter
f_to_decimate	200 Hz	Frequency to which the percentile-filtered trace was downsampled to find the baseline
Median filter order	3 points	Median filter width applied to the 200 Hz signal
$\widehat{SD}$	1.4826×MAD	Standard deviation was estimated robustly using this formula, where MAD is the median absolute deviation around the median.
Deconvolution parameters (OASIS)		
noise_factor	0.8	Noise is this factor × $\widehat{SD}$
τ_{decay}	3.5 ms	Decay time constant of the kernel
τ_{rise}	0.7 ms	Rise time constant of the kernel
Kernel formula	$n \times exp(-t/\tau_{decay}) \times (1 - exp(-t/\tau_{rise}))$	where n normalizes the peak to 1
max_iter	10	Number of iterations OASIS algorithms runs for
penalty	1	1 means it uses the L1 penalty to sparsely reconstruct the signal.
oasis.functions.deconvolve		The function in the OASIS library used for the deconvolution
Peak detection parameters		
noise_factor	0.5	Minimum peak height and prominence is this factor × $\widehat{SD}$
split_gap_ms	2.0 ms	Minimum gab between two peaks
conv_filt_ms	2.5 ms	Width of the triangular filter used on the deconvolved trace
scipy.signal.find_peaks		The function used to find peaks in the deconvolved trace
Segmentation (clustering) parameters		

thresh	1.8	This factor × $\widehat{SD}$ is the height below which the denoised trace must go to split it. Lower thresh leads to more events per cluster.
pre_c_ms	10 ms	The previous cluster is combined if it is within this distance when it falls below the threshold
post_c_ms	20 ms	The next cluster is combined if it is within this distance when it falls below the threshold
pre_j_ms	10 ms	The previous cluster is combined if it is within this distance of the first event
post_j_ms	10 ms	The next cluster is combined if it is within this distance of the last event
Fitting parameters		
jitter	10 ms	How far the event onset time o can be fit from the initial estimate $\hat{o}$
height_factor_high	3	how many bigger times the event height h can be fit from the initial estimate $\hat{h}$
height_factor_low	15	how many smaller times the event height h can be fit from the initial estimate $\hat{h}$
tau1_min	0.5 ms	Lower bound of τ_{decay}
tau1_max	50 ms	Upper bound of τ_{decay}
tau2_min	0.1 ms	Lower bound of τ_{rise}
tau2_max	10 ms	Upper bound of τ_{rise}
noise_factor	1	The root mean square error between the signal and its reconstruction $[RMS(S - \tilde{s})]$ is divided by noise_factor×$\widehat{SD}$ to normalize it.
regularization	0.2	The root mean square error between the onset time and its estimate $[RMS(o - \hat{o})]$ is multiplied by this number to get the regularization. Using these values of noise_factor and regularization yields $\frac{RMS(S-\tilde{s})}{\widehat{SD}} + \frac{RMS(o-\hat{o})}{5}$ which is being minimized.
maxiter_DA	5000×num_events_in_cluster	num_events_in_cluster is the number of events in the cluster

maxfev_DA	5000×sqrt(num_events_in_cluster)	
maxiter_Powell	300×num_events_in_cluster	
maxfev_Powell	3000×num_events_in_cluster	

Table S5. Priors used in the Bayesian connectivity models.

Prior	Value	Comments
evoked_window	90 ms	Disregarding the last 10 ms before the next stimulation
rate_mu	4.83 Hz	Calculated empirically from the distribution of rates during spontaneous intervals over all cells
rate_sigma	3.79 Hz	
Evoked_per_trial_mu	1.0	An informative prior; these values define a connected cell in the model assuming connection (model 2)
Evoked_per_trial_sigma	0.35	
bump_center_mu	12.7 ms	Calculated empirically from the data used in Figure 2b
bump_width_mu	6.33 ms	
bump_center_sigma	6.33 ms	
bump_width_sigma	1.5 ms	A small value to for computational stability
