# Supplementary material for: Determinants of functional synaptic connectivity among amygdala-projecting prefrontal cortical neurons in male mice
Source: Nat Commun. 2023 Mar 25;14:1667. doi: 10.1038/s41467-023-37318-x (PMC10039875; doi:10.1038/s41467-023-37318-x)
Supplement: Supplementary file 3 — Reporting Summary [file 41467_2023_37318_MOESM3_ESM.pdf]

Corresponding author(s): Ofer Yizhar

Last updated by author(s): Jan 9, 2023

Reporting Summary

Nature Portfolio wishes to improve the reproducibility of the work that we publish. This form provides structure for consistency and transparency in reporting. For further information on Nature Portfolio policies, see our Editorial Policies and the Editorial Policy Checklist.

Statistics

For all statistical analyses, confirm that the following items are present in the figure legend, table legend, main text, or Methods section.

n/a	Confirmed	
☐	☒	The exact sample size (*n*) for each experimental group/condition, given as a discrete number and unit of measurement
☐	☒	A statement on whether measurements were taken from distinct samples or whether the same sample was measured repeatedly
☐	☒	The statistical test(s) used AND whether they are one- or two-sided<br>*Only common tests should be described solely by name; describe more complex techniques in the Methods section.*
☐	☒	A description of all covariates tested
☐	☒	A description of any assumptions or corrections, such as tests of normality and adjustment for multiple comparisons
☐	☒	A full description of the statistical parameters including central tendency (e.g. means) or other basic estimates (e.g. regression coefficient) AND variation (e.g. standard deviation) or associated estimates of uncertainty (e.g. confidence intervals)
☐	☒	For null hypothesis testing, the test statistic (e.g. *F*, *t*, *r*) with confidence intervals, effect sizes, degrees of freedom and *P* value noted<br>*Give P values as exact values whenever suitable.*
☐	☒	For Bayesian analysis, information on the choice of priors and Markov chain Monte Carlo settings
☐	☒	For hierarchical and complex designs, identification of the appropriate level for tests and full reporting of outcomes
☐	☒	Estimates of effect sizes (e.g. Cohen's *d*, Pearson's *r*), indicating how they were calculated

Our web collection on statistics for biologists contains articles on many of the points above.

Software and code

Policy information about availability of computer code

Data collection	Prairie View version 5.5 was used for two-photon optogenetic stimulation and collection of two-photon imaging data. Custom scripts written in Matlab version R2018b were used to generate two-photon scan patterns that were imported into Prairie View. Electrophysiological data were acquired using pClamp 10.4.1.10 (Molecular Devices). VS-ASW 2.9 (Olympus) was used for image acquisition on the Olympus VS120 slide-scanning microscope, and ZEN 14.0 (Zeiss) was used to acquire images on the Zeiss LSM 700 confocal microscope.
Data analysis	The following software were used for data analysis: Python version 3.8.5, Matlab version R2018b, Fiji (ImageJ) version 1.53c, and Clampfit version 11.0.3.03. Details on the specific Python and Matlab packages and functions that were used, including the Python ArviZ package, are provided in the relevant Methods sections of the manuscript. Analysis codes will be made openly available toward publication or upon request from reviewers.

For manuscripts utilizing custom algorithms or software that are central to the research but not yet described in published literature, software must be made available to editors and reviewers. We strongly encourage code deposition in a community repository (e.g. GitHub). See the Nature Portfolio guidelines for submitting code & software for further information.

Data

Policy information about availability of data

All manuscripts must include a data availability statement. This statement should provide the following information, where applicable:

- Accession codes, unique identifiers, or web links for publicly available datasets
- A description of any restrictions on data availability
- For clinical datasets or third party data, please ensure that the statement adheres to our policy

Data generated and used in this study will be made available in a publicly accessible data repository. For this study, a reference coronal mouse brain atlas was used (https://kimlab.io/brain-map/atlas/).

Human research participants

Policy information about studies involving human research participants and Sex and Gender in Research.

Reporting on sex and gender	*Use the terms sex (biological attribute) and gender (shaped by social and cultural circumstances) carefully in order to avoid confusing both terms. Indicate if findings apply to only one sex or gender; describe whether sex and gender were considered in study design whether sex and/or gender was determined based on self-reporting or assigned and methods used. Provide in the source data disaggregated sex and gender data where this information has been collected, and consent has been obtained for sharing of individual-level data; provide overall numbers in this Reporting Summary. Please state if this information has not been collected. Report sex- and gender-based analyses where performed, justify reasons for lack of sex- and gender-based analysis.*
Population characteristics	*Describe the covariate-relevant population characteristics of the human research participants (e.g. age, genotypic information, past and current diagnosis and treatment categories). If you filled out the behavioural & social sciences study design questions and have nothing to add here, write "See above."*
Recruitment	*Describe how participants were recruited. Outline any potential self-selection bias or other biases that may be present and how these are likely to impact results.*
Ethics oversight	*Identify the organization(s) that approved the study protocol.*

Note that full information on the approval of the study protocol must also be provided in the manuscript.

Field-specific reporting

Please select the one below that is the best fit for your research. If you are not sure, read the appropriate sections before making your selection.

☒ Life sciences ☐ Behavioural & social sciences ☐ Ecological, evolutionary & environmental sciences

For a reference copy of the document with all sections, see nature.com/documents/nr-reporting-summary-flat.pdf

Life sciences study design

All studies must disclose on these points even when the disclosure is negative.

Sample size	No statistical tests were run to predetermine sample sizes. Our sample sizes were based on standards in the field, such as in ref. 22 (Cossell et al., Nature 2015).
Data exclusions	Stimulated cells (candidate presynaptic cells) whose GCaMP6s signal did not cross a predetermined threshold, which is described in detail in the Methods section, were excluded from analysis so that their lack of spiking is not mistaken for lack of synaptic connection to the corresponding recorded (postsynaptic) cell.
Replication	Upon collection of approximately half of the data, intermediate analyses of connectivity were performed and yielded similar main findings to those obtained from analysis of the entire dataset, which are reported in the manuscript. After the intermediate analyses, the next connectivity analyses were performed after collection of the entire dataset.
Randomization	C57BL6 mice were allocated to groups by the virus injected to them. All injections performed for connectivity mapping were done into the left hemisphere. Mice expressing stCoChR and GCaMP6s in mPFC-BLA cells were used for recording from mPFC-BLA cells as well as for recording from non-mPFC-BLA cells. Mice expressing stCoChR and GCaMP6s sparsely in the mPFC were used for recording from random mPFC cells.
Blinding	Investigators could not be blinded to group allocation during data collection since the recordings were performed from cells based on their expression. Most of the analysis process could not be blinded as well since photocurrents could be evoked in stCoChR-expressing cells, which were visible during analysis of their connectivity. However, during the use of the Bayesian connectivity-analysis model, the experimenters were blinded to cell identity.

Reporting for specific materials, systems and methods

We require information from authors about some types of materials, experimental systems and methods used in many studies. Here, indicate whether each material, system or method listed is relevant to your study. If you are not sure if a list item applies to your research, read the appropriate section before selecting a response.

Materials & experimental systems

n/a	Involved in the study
☐	☒ Antibodies
☐	☒ Eukaryotic cell lines
☒	☐ Palaeontology and archaeology
☐	☒ Animals and other organisms
☒	☐ Clinical data
☒	☐ Dual use research of concern

Methods

n/a	Involved in the study
☒	☐ ChIP-seq
☒	☐ Flow cytometry
☒	☐ MRI-based neuroimaging

Antibodies

Antibodies used	Polyclonal rabbit anti-GFP primary antibody (ThermoFisher Scientific, catalog # A-11122); polyclonal goat anti-rabbit secondary antibody conjugated to Alexa Fluor 488 (Abcam, catalog # ab150077)
Validation	Validation was performed on primary hippocampal neuronal cultures to verify specific binding in neurons expressing GCaMP vs. non-expressing control neurons.

Eukaryotic cell lines

Policy information about cell lines and Sex and Gender in Research

Cell line source(s)	HEK293 cells were used for production of recombinant AAV vectors.
Authentication	The cell line used was not authenticated.
Mycoplasma contamination	The cell line was not tested for mycoplasma contamination.
Commonly misidentified lines (See ICLAC register)	No commonly misidentified lines were used in the study.

Animals and other research organisms

Policy information about studies involving animals; ARRIVE guidelines recommended for reporting animal research, and Sex and Gender in Research

Laboratory animals	Male C57BL6 mice aged 1-5 months (and one aged 9.6 months), and male Ai9 mice aged 1-2 months were used in this study.
Wild animals	No wild animals were used in this study.
Reporting on sex	The findings of this study apply only to males as only male mice were used.
Field-collected samples	No field-collected samples were used in this study.
Ethics oversight	All procedures described in this manuscript were approved by the Weizmann Institute Animal Care and Use Committee (IACUC).

Note that full information on the approval of the study protocol must also be provided in the manuscript.
